# Co-developing sleep-wake and sensory foundations for cognition in the human fetus and newborn

**DOI:** 10.1016/j.dcn.2024.101487

**Published:** 2024-12-12

**Authors:** Kimberley Whitehead

**Affiliations:** Research Division of Digital Health and Applied Technology Assessment (DHATA), Florence Nightingale Faculty of Nursing, Midwifery & Palliative Care, King’s College London, James Clerk Maxwell Building, 57 Waterloo Rd, London SE1 8WA, UK

**Keywords:** Neonate, Infant, Behaviour, Motor, Intrauterine growth restriction

## Abstract

In older children and adults, cognition builds upon waking sensory experience which is consolidated during sleep. In the fetus and newborn, sensory input is instead largely experienced *during* sleep. The nature of these sensory inputs differs within sleep, between active and quiet sleep, as well as versus wakefulness. Here, sleep-wake organisation in the fetus and newborn is reviewed, and then its interaction with sensory inputs discussed with a focus on somatosensory and auditory modalities. Next, these ideas are applied to how neurological insults affect early development, using fetal growth restriction as a test case. Finally, the argument is made that taking account of sleep-wake state during perinatal functional neuroimaging can better index sensorimotor, language, and cognitive brain activities, potentially improving its diagnostic and prognostic value. To sum up, sensory and sleep-wake functions go hand in hand during early human development. Perturbation of these twinned functions by neurological insults may mediate later neurodevelopmental deficits. Perinatal neuroimaging has the potential to track these trajectories, feasibly identifying opportunities to therapeutically intervene.

## Highlights

• Sensory inputs are organised by sleep-wake state in the fetus and newborn.
• Sleep-wake and sensory processing foundations for cognition are intertwined.
• Perinatal brain insults perturb both foundations.
• Perinatal functional neuroimaging can beneficially account for sleep-wake state.

## Graphical Abstract

**Figure:**
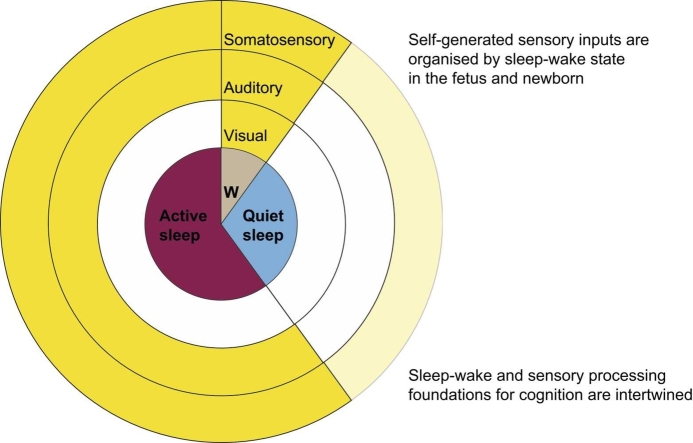

## 1 Introduction

In older children and adults, cognition builds upon waking sensory experience which is consolidated during sleep (Buzsáki, 1989). In the fetus and newborn, sensory input is instead largely experienced *during* sleep. This sleep can be broadly categorised as active sleep (precursor to rapid eye movement (REM) sleep) or quiet sleep (precursor to non-REM sleep). This review focuses on interactions between sleep-wake and sensory functions during the unique perinatal period, before a switch point to adult-like features at 2–3 months of age which include circadian rhythmicity, REM sleep muscle atonia, non-REM onset sleep, and mature cortical signatures of sleep-wake state (e.g. spindles during non-REM sleep, alpha and mu rhythms during wakefulness) (Grigg-Damberger, 2016, Jenni et al., 2006, Kohyama, 1996, Saby and Marshall, 2012, Sokoloff et al., 2021). It is argued here that perinatal sleep-wake organisation cannot be fully appreciated isolated from its sensory correlates. Next, the case is made that perinatal brain insults perturb twinned sleep-wake and sensory functions, and that accounting for this deepens understanding of how insults impact early development.

## 2 Sleep-wake state organisation in the human fetus and newborn

Rudimentary behavioural cycling emerges in the fetus during the final trimester of gestation from an ‘indeterminate’ state to well-defined active and quiet periods. During active states, movement patterns include limb and rapid eye movements, respiratory ‘breathing’ movements, and hiccups (Gazzolo et al., 1995, Groome et al., 1997a, Shuffrey et al., 2019, Ten Hof et al., 2002). During the quiet state, the fetus is almost completely quiescent. In parallel, heart rate variability is relatively high during active states and lower in the quiet state (Nijhuis et al., 1982, de Vries and Fong, 2006, Zavala et al., 2020).

**Fig. 1:**
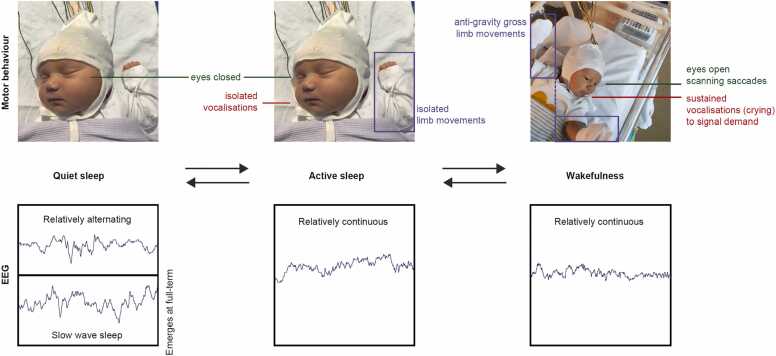
**Motor behaviour (upper panel) and cortical activity (lower panel) during sleep-wake states.** Electroencephalography (EEG) examples are from an infant aged 42 corrected gestational weeks (Whitehead et al., 2018a).

Postnatally, active states can be subdivided into wakefulness and active sleep, with quiet sleep occupying the remainder. Example proportions in an early-term neonate are approximately 10 % wakefulness, 60 % active sleep, and 30 % quiet sleep (Graphical Abstract) (Georgoulas et al., 2021, Groome et al., 1997b, Roffwarg et al., 1966). Both active states are associated with relatively continuous low-amplitude cortical activity, while quiet sleep is characterised by relatively alternating high-amplitude cortical activity (Fig. 1). State-organised motor behaviour continues, as during the fetal period, and now also incorporates vocalisations, and scanning open eye movements when awake (Fig. 1). As in the fetus, heart rate variability is higher during active states than quiet sleep (Isler et al., 2016), meaning that this index of state is continuous from fetal to neonatal data, facilitating their integration.

## 3 Self-generated sensory inputs across sleep-wake states

Spontaneous motor behaviour self-generates sensory inputs. Every movement, vocalisation, and open eye movement will generate incoming somatosensory, auditory, and visual input respectively. Because these motor behaviours are organised across the sleep-wake cycle (Fig. 1), so too is self-generated sensory input (Graphical Abstract).

### 3.1 Somatosensory inputs

**Fig. 2:**
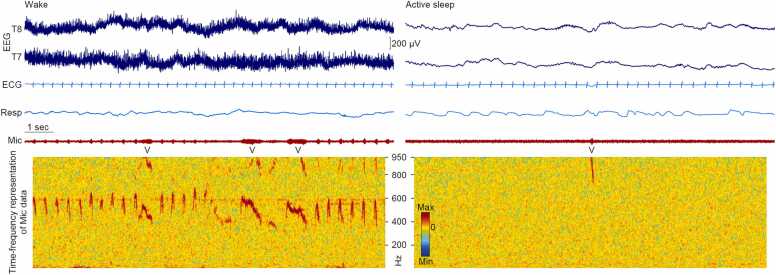
**Vocalisations during wakefulness and active sleep.** Examples of vocalisations (‘V’) recorded with a collar-affixed microphone (Unimed Electrode Supplies, Mic), and their time-frequency representation (EEGLAB v. 2024.2), time-locked to EEG at temporal channels T8 and T7 which overlie auditory cortex, ECG, and respiratory movement (Resp). Left: Infant aged 35 corrected gestational weeks exhibits a sequence of cries during wakefulness. Note the sustained electromyographic artefact on the EEG channels, consistent with wakefulness. Right: Infant aged 35 corrected gestational weeks exhibits an isolated ‘squeak’ during active sleep (associated to Supplementary Audio File). Note the lack of sustained electromyographic artefact, consistent with sleep. All data were sampled at 2000 Hz. EEG data were high-pass filtered at 0.3 Hz and baseline corrected.

Spontaneous movements evoke incoming somatosensory input, via proprioceptive feedback as the body moves through space and/or via tactile inputs from the body brushing its surroundings. These movements vary between sleep-wake states. During quiet sleep, long periods of behavioural quiescence are interrupted by whole-body reflexive ‘startles’ (de Groot et al., 2022, Hadders-Algra et al., 1993) and, more subtly, discrete respiratory movement events comprising large-magnitude sighs (Edwards et al., 2018, Fleming et al., 1984). During active sleep, movements are profuse, with isolated limb movements a hallmark of the motor repertoire, which cluster alongside rapid eye movements (Curzi-Dascalova et al., 1993, Hadders-Algra et al., 1993, Kohyama, 1996, Sokoloff et al., 2020, Sokoloff et al., 2021, Whitehead et al., 2019c, Whitehead et al., 2020); Fig. S2 in (Koskela et al., 2021). Respiratory movement events are also prominent, with large-magnitude sighs interspersed with periods of shallow breathing (hypopnea), and variations in breathing frequency (Fig. 2) (Shiraki et al., 2024). Wakefulness is characterised by gross and largely continuous, rather than isolated, body movements, although these appreciably wax and wane (Hadders-Algra et al., 1992, Hadders-Algra et al., 1993), and some behavioural scoring systems use this variance to distinguish ‘active’ from ‘quiet’ wakefulness (e.g. (Stevens et al., 2014)). Respiratory movement patterning during wakefulness is comparable to active sleep, although extremely large-magnitude events – hiccups – are more prevalent (Brouillette et al., 1980, Whitehead et al., 2019b).

Taken together, movement varies between states but also fluctuates *within*-states, effectively representing an additional layer of intra-state organisation. This behaviour is tracked by the neonate’s brain activity, because spontaneous movements evoke cortical responses, even during sleep. These ∼1 second duration responses are somatotopic, i.e. correspond to the way that the body is represented within sensorimotor cortex. For example, symmetrical movements, like startles and hiccups, evoke responses with a midline central distribution (Losito et al., 2017, Whitehead et al., 2019b), while unilateral hand movements evoke responses with a contralateral central distribution (Allievi et al., 2016, Milh et al., 2007, Vanhatalo et al., 2009, Whitehead et al., 2018b). In sum, sleep-wake state is the organisational backbone of perinatal motor activity, which evokes somatotopic brain responses at a time of rapid body map functional development (Allievi et al., 2016, Dall’Orso et al., 2018, Whitehead et al., 2019a). These body maps are proposed to underlie infants’ distinction of ‘self’ versus ‘other’: a crucial prerequisite for social cognition (Marshall and Meltzoff, 2015).

### 3.2 Auditory inputs

An infant’s vocalisations provide incoming *auditory* input to their developing brain, and are likewise organised across sleep-wake states. In quiet sleep, vocalisations are absent. In active sleep, vocalisations are present as intermittent grunts, cries and squeaks (Fig. 2 and Supplementary Audio File) (de Groot et al., 2022). During wakefulness, vocalisations fall into three categories. First, they are present as comparable intermittent vocalisations to those observed during active sleep, but here termed ‘protophones’, meaning precursors to speech (Oller et al., 2019, Zhang and Ghazanfar, 2016). Second, other sections of wakefulness are dominated by sustained crying, although with a clear embedded temporal structure (Fig. 2) (Cabana et al., 2021, Sahni et al., 1995). Third, so-called ‘vegetative’ vocalisations include the self-generated sound caused by abrupt closure of the glottis during hiccups, and burps and sneezes (Oller et al., 2019). Like somatosensory inputs, auditory inputs evoke brain responses in the neonate (Chipaux et al., 2013, Dall’Orso et al., 2021, Fifer et al., 2010, Kaminska et al., 2018), although these have not been specifically studied after self-generated stimuli except hiccups (Whitehead et al., 2019b).

As with self-generated somatosensory input then, self-generated auditory input varies both between and within-states. Parallels include active sleep being characterised by both movements and vocalisations which are discrete and isolated (Fig. 2), while wakefulness is marked by ‘noisier’ and more continuous sensory inputs. Nevertheless, even apparently ‘noisy’ signals like crying, contain temporal structure (Fig. 2). The auditory system is built on the organisational system of tonotopy, with sounds of different frequency spatially represented across auditory cortex, just as the somatosensory system is built on that of somatotopy. Therefore, the varied and multi-frequency vocalisations across sleep and wakefulness may be well-suited to refine cortical encoding of this range, which subserves recognition of and attendance to human speech, also important for social cognition (Oller et al., 2019).

### 3.3 Visual inputs

During either quiet or active sleep, the closed eyelids mean that only gross external visual inputs – like flashes – evoke cortical responses (Colonnese et al., 2010). Wakefulness – during which the eyes are open and exhibit scanning saccadic movements – is the only state to provide patterned visual input corresponding to the external world, albeit low resolution, to the developing cortex (Fantz, 1963, Miranda, 1970, Wolff, 1965).

### 3.4 Summary of sensory inputs across sleep-wake states

Drawing this section together, each sleep-wake state has a signature of motor behaviour and associated somatosensory, auditory, and visual self-generated input (Fig. 1 and Graphical Abstract), at a time when the developing brain is beginning to exhibit multimodal sensory learning (Dall’Orso et al., 2021, Fifer et al., 2010). Further to motor behaviours self-generating sensory inputs, they also provoke supportive sensory caregiving activities, creating early conditioning associations between self-actions and positive results. Gross movements, crying, and eye opening during wakefulness likely contribute to the short latency to being picked up upon awakening (Fig. S4 in (Georgoulas et al., 2021)). The more subtle motor activity and vocalisations of active sleep may also trigger sensory interventions, given that longer active sleep bouts are associated with higher frequency of caregiver contacts (Bowe and Anders, 1979). In fact, it is likely that active sleep is regularly mistaken for wakefulness, given that even neonatal healthcare staff can rarely distinguish them behaviourally, partly owing to lack of awareness that sustained eye opening is an important signature of wakefulness (de Groot et al., 2023)

## 4 Sleep organisation in the fetus and newborn affected by a neurological insult

As described above, the human fetus exhibits a repertoire of motor behaviour including limb movements and hiccups. The importance of this motor behaviour is emphasised by it being an index of perinatal neurological health. Fetal limb and respiratory ‘breathing’ movements positively correlate with oxygen and glucose levels (Bocking et al., 1982, Vasung et al., 2022), and fewer fetal movements and hiccups predicts stillbirth, or adverse neurodevelopment in survivors (Thompson et al., 2021, Zamstein et al., 2019). However, these data are rarely interpreted using the lens of sleep.

**Fig. 3:**
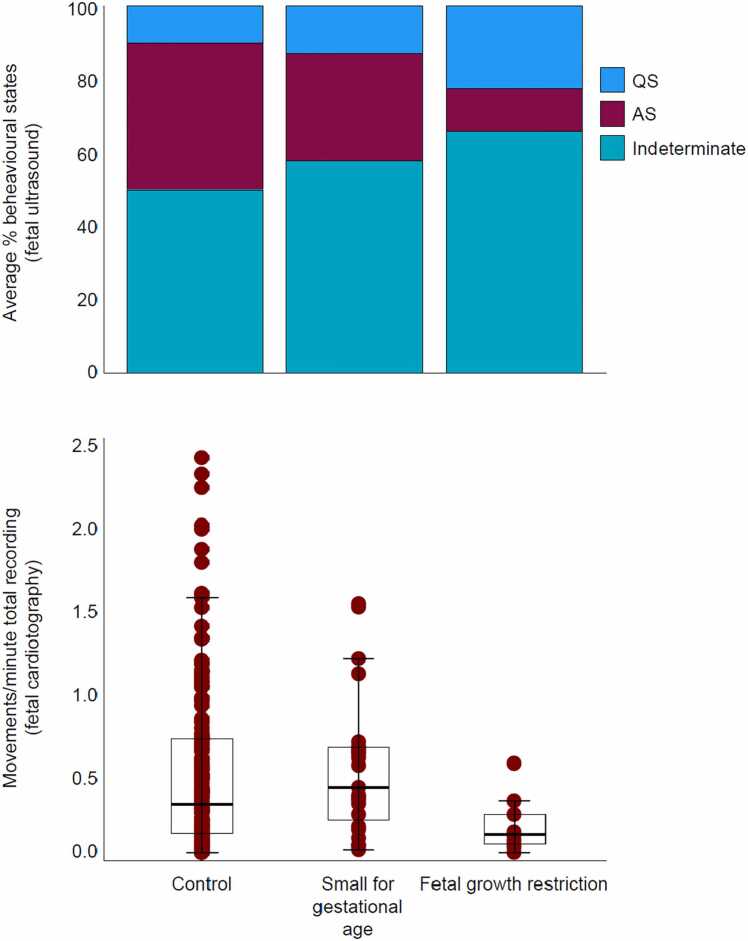
Intrauterine **growth restriction is associated with reduced active sleep and reduced movements antenatally.** Upper panel: data sourced from (Gazzolo et al., 1995). Lower panel: data sourced from (Higgins et al., 2018). Please see Supplementary Methods and Supplementary Spreadsheet for details. QS = Quiet Sleep; AS = Active Sleep.

A form of fetal compromise often associated with reduced fetal movements is intrauterine growth restriction secondary to placental insufficiency (Gordijn et al., 2016, Higgins et al., 2018, Lucchini et al., 2021, Mainous and Hueston, 1994). Unlike constitutionally small fetuses – termed ‘small for gestational age’ – growth-restricted fetuses are small because of chronic hypoxemia and hypoglycaemia in utero. It is often noted that these fetuses likely stop moving to minimise expenditure of their limited resources. Combining these observations with fetal sleep data implies that at least part of the attenuation in motor activity could be associated with reduced active sleep (Fig. 3) (also observed in a sheep model of the condition (Keen et al., 2011)). Like motor activity, active sleep is metabolically demanding, with oxygen consumption higher than in quiet sleep (Azaz et al., 1992, Scopes and Ahmed, 1966, Stabell et al., 1977, Stothers and Warner, 1978). In line with this, active sleep accentuates the fetal heart rate abnormalities characteristic of intrauterine growth restriction (Sriram et al., 2013).

While reduced motor activity and active sleep may be advantageous to the compromised fetus in the short-term, both are thought to be necessary for appropriate sensorimotor and cognitive development based on theoretical and animal models (Blumberg et al., 2020, Cao et al., 2016, Frank et al., 2001, Jha et al., 2005). Consequently, the adverse motor, language and cognitive outcomes observed in survivors of fetal growth restriction could result not only from the insult itself, but these adaptations (Lingam et al., 2023, Sacchi et al., 2021). This might explain why those affected display *more* active sleep during the neonatal period once they have left the constraints of the uterine environment, in an attempt to counteract its earlier shortage (Hoppenbrouwers et al., 2005).

In many cases, the effects of intrauterine growth restriction on antenatal sleep are then compounded by the effects of premature birth on postnatal sleep, because affected pregnancies often require medically-indicated preterm delivery (Lingam et al., 2023). These include that some preterm infants exhibit relatively little differentiation between cortical activity in each sleep-wake state, which predicts adverse sensorimotor and cognitive outcomes (Conde et al., 2005, El-Dib et al., 2014, Hellström-Westas et al., 1991, Tokariev et al., 2019, Weisman et al., 2011, Wikström et al., 2012).

Finally, preterm infants born after intrauterine growth restriction require particularly long neonatal intensive care unit (NICU) admissions (Lingam et al., 2023). This means that the disruptive effects on sleep of i) intrauterine growth restriction, and ii) prematurity, are finally exacerbated by iii) extended disturbance associated to the sensory environment of the NICU. Clinically necessary painful and other care procedures, and physiological stress, evoke and extend awakenings, especially from the late preterm period onwards (Georgoulas et al., 2021, Grunau et al., 2000, Holditch-Davis et al., 1995). This means that sleep disturbance could be a mediator of the reported association between pain experience in preterm infants and abnormal neurological outcomes (Brummelte et al., 2012, Maitre et al., 2017, Ranger et al., 2013). In summary, in the test case of an infant who suffers intrauterine growth restriction, is born preterm, and then spends weeks on the NICU, all three experiences are likely to contribute to suboptimal sleep.

## 5 Accounting for sleep-wake state during neonatal functional neuroimaging

The literature reviewed up to now shows that sleep interacts with sensory, language and cognitive brain development. Hence, there are advantages to accounting for sleep-wake state during functional neuroimaging of the young brain. Indeed, unlike in adult neuroimaging, when the subject is almost exclusively awake, fetal and neonatal neuroimaging almost always occurs during natural sleep, because this is so prevalent (Georgoulas et al., 2021). Particularly relevant for the sensory experimental paradigms typically used in this immature population, sleep state affects how sensory input is processed from periphery to cortex. For the visual system, the neonatal retina exhibits higher amplitude evoked responses during active than quiet sleep (Peña et al., 1999), and visual cortical potentials are more reliably elicited in active sleep too (Cubero-Rego et al., 2018). On the other hand, somatosensory and auditory cortical responses are higher amplitude during quiet than active sleep, particularly for higher-order potentials (beyond primary sensory cortex) (Desmedt and Manil, 1970, Kaminska et al., 2018, Nevalainen et al., 2008, Pihko et al., 2004).

While the results discussed above suggest the value of modelling the effect of sleep-wake state on functional neuroimaging data, this can be challenging in developmental populations. Solutions applicable to fetal functional magnetic resonance imaging (fMRI) data include to leverage the timings of movement-related artefact (Ji et al., 2023). In *neonates* undergoing fMRI, strategies could also utilise the associated cardiorespiratory data (recorded during the scan for safety reasons), especially if synchronised EEG could provide a ‘ground truth’ reference point in at least a small population (Arichi et al., 2017). Similarly, in near infra-red spectroscopy (NIRS) recordings, behavioural sleep-wake staging can be strengthened with concurrent EEG (Gossé et al., 2023, Lee et al., 2020, Shellhaas et al., 2014, Shiraki et al., 2024). Other options include to *account* for sleep state, even if not treating it as a variable of interest (Colonnese et al., 2010, Whitehead et al., 2019a), separate data into quiet versus active states (pooling active sleep and wakefulness, given their comparable cortical activity (Fig. 1)) (Bongers-Schokking et al., 1989, Montazeri et al., 2022b), or pragmatically treat sleep as an overall state in the youngest preterm subjects for whom active and quiet sleep are less well-defined (Whitehead et al., 2022).

Taking a wider view, accounting for sleep could help to address the larger issue in science of reproducibility failures, in conjunction with greater availability of open-access sleep data and algorithms. For example, open-access sleep-staged EEG and associated data from preterm and older infants are available via (Blumberg and Sokoloff, 2021, Jones et al., 2018, Lee et al., 2022, Whitehead and Fabrizi, 2024). Further, two open-access EEG datasets (not originally associated to sleep stages) have now been sleep scored: the (Stevenson et al., 2019) dataset by (Tamburro et al., 2022), and the (O’Toole et al., 2022) dataset by (Montazeri et al., 2022a). The sleep scoring algorithm employed by the latter, and its visualization tools, can be used openly via a cloud service by requesting access from the authors (Montazeri et al., 2022b). Maternal-fetal open-access resources are rarer, but an interesting recently released example integrates fetal heart rate data with maternal sleep staging (DiPietro et al., 2021, DiPietro et al., 2023, DiPietro, 2023).

## 6 Conclusion

In early human development, sensory and sleep-wake functions go hand in hand. Perturbation of these twinned functions by neurological insults may mediate later sensorimotor, language and cognitive deficits. Perinatal neuroimaging modalities have the potential to track these trajectories, offering diagnostic and prognostic value, as well as identifying opportunities to therapeutically intervene. Promoting of this, developmental sleep neurology is a burgeoning research field, widely supported by families and stakeholders (de Groot et al., 2023, van den Hoogen et al., 2017, Whitehead, 2024).

## CRediT authorship contribution statement

**Kimberley Whitehead:** Writing – original draft.

## Declaration of Competing Interest

The author declares that they have no known competing financial interests or personal relationships that could have appeared to influence the work reported in this paper.

## Data Availability

Open-access data were used to generate Fig. 3: please see Supplementary Methods and Supplementary Spreadsheet for details.

## Footnotes

Appendix A Supplementary data associated with this article can be found in the online version at doi:10.1016/j.dcn.2024.101487.
