# Supplementary material for: Co-developing sleep-wake and sensory foundations for cognition in the human fetus and newborn
Source: Dev Cogn Neurosci. 2024 Dec 12;71:101487. doi: 10.1016/j.dcn.2024.101487 (PMC11699341; doi:10.1016/j.dcn.2024.101487)
Supplement: Supplementary file 1 — Supplementary material [file mmc1.docx]

**How Fig. 3 was generated**

**Fetal behavioural states panel of Fig. 3**

Individual-level data on small fetuses were pulled from Table 1 of [Gazzolo et al., 1995]. To distinguish fetal growth restriction from constitutionally small fetuses (small for gestational age), the former were defined here as exhibiting fetal distress (FD) (a column of Table 1). The data for appropriate size for gestational age control fetuses was reported as a group-level average in Table 3 of [Gazzolo et al., 1995], and is named ‘GazzControlmedian’ in the Supplementary Spreadsheet. Data are plotted from time point 1 (27-32 weeks gestational age) because there are more data available here than time point 2. The proportion of active sleep (termed in [Gazzolo et al., 1995] ‘coincident state 2F’) is lower in fetal growth restriction than fetuses only small for gestational age (p = .022, unpaired t test, other states p >.05, IBM SPSS Statistics 26). It is not possible to statistically compare control fetuses to the other groups, because only a group-level average is available for the former.

**Fetal movements panel of Fig. 3**

Individual-level data were sourced from [Higgins et al., 2018]. Fetal growth restriction was defined as fetal or neonatal weight below the 3^rd^ centile and/or static fetal growth with fetal or birth weight below the 10^th^ centile [Gordijn et al., 2016]. Small for gestational age was defined as birth weight below the 10^th^ centile, in the absence of meeting any criteria for fetal growth restriction. Appropriate size for gestational age controls were defined as birth weight above the 15^th^ centile. Data excluded comprised two pregnancies which did not result in a live birth after placental abruption, subject RFM197 because a discrepancy appeared to reflect a coding error in the original [Higgins et al., 2018] spreadsheet, and infants whose birth weight fell between the 10^th^-15^th^ centile in the absence of meeting criteria for fetal growth restriction. Movement rate was lower in fetal growth restriction than either controls or fetuses only small for gestational age (p = <.001 and p = .002 respectively, unpaired t test).

**Spreadsheet associated to Fig. 3**

Please see Supplementary Spreadsheet for the data used to generate Fig. 3.

**References**

Gazzolo D, Visser GHA, Santi F, Magliano CP, Scopesi F, Russo A, Pittaluga C, Nigro M, Camoriano R, Bruschettini PL (1995): Behavioural development and Doppler velocimetry in relation to perinatal outcome in small for dates fetuses. Early Hum Dev 43:185–195.

Gordijn SJ, Beune IM, Thilaganathan B, Papageorghiou A, Baschat AA, Baker PN, Silver RM, Wynia K, Ganzevoort W (2016): Consensus definition of fetal growth restriction: a Delphi procedure. Ultrasound Obstet Gynecol 48:333–339.

Higgins L, Johnstone E, Heazell A (2018): FEMINA2 1. https://data.mendeley.com/datasets/vsxsjhp6n3/1.
